# Undergraduate nursing students’ experiences during international clinical placement: A scoping review

**DOI:** 10.1016/j.ijnsa.2025.100378

**Published:** 2025-07-16

**Authors:** Lasse Andreassen, Simen A. Steindal, Jyoti Sarin, Benedicte Sørensen Strøm

**Affiliations:** a Akershus University Hospital, HF, P.O. Box 1000, Lørenskog 1478, Norway; b Institute of nursing, VID Specialized University, Oslo, Norway; c Lovisenberg Diaconal University College, Oslo, Norway; d Maharishi Markendeshwar College of Nursing, Maharishi Markendeshwar (Deemed to be University), Mullana, Ambala, Haryana 133207, India

**Keywords:** International clinical placement, Nursing education, Nursing students, Review

## Abstract

**Background:**

Immigration, combined with the growth of multicultural societies, has increased patient diversity, highlighting the need for cultural competence in healthcare to prepare nurses to understand the nature of global health. Nurses should have knowledge to understand and respect how culture influences patients’ health needs, access to healthcare services, and the health-related decision-making process. Offering international clinical placement to nursing students is one way to increase this cultural competence. No scoping review has mapped undergraduate nursing students’ experiences during international clinical placement.

**Objectives:**

To provide an overview of published studies on undergraduate nursing students’ experiences during international clinical placement.

**Design:**

A scoping review.

**Methods:**

A systematic search was performed in three databases (CINAHL, Medline, PubMed). The inclusion criteria were: 1) studies with quantitative, qualitative or mixed methods design, 2) studies reporting undergraduate nursing students’ experiences of international clinical placement and 3) studies published in peer-reviewed journals in the English language between January 2008 and February 2023. Pairs of authors independently assessed eligibility and extracted data. Data were thematically grouped.

**Results:**

42 papers were included, 39 studies employed a qualitive design, while 3 studies employed a quantitative design. All studies included students from Western countries with international clinical placement in non-Western countries. Most students had international clinical placement in hospitals, with a wide range of length of exchange. Four thematic groupings were identified: 1) developing cultural competence through culture shock, 2) a novel way of practicing nursing due to a different nursing role, 3) the essential role of the supervisor in learning outcomes and 4) the impact of language barriers on learning outcomes.

**Conclusions:**

Students developed cultural competence by experiencing culture shock when exposed to an unfamiliar cultural environment. While they often reacted negatively to these differences, they often adjusted their prejudices and biases over time. Language skills were the greatest barrier to achieving learning outcomes, creating more frustration than cultural barriers. There is a need to investigate how non-Western students experience studying in a Western country.

**What is already known about the topic?**

• Immigration, combined with the growth of multicultural societies, has increased patient diversity
• There is an increasing need for cultural competence in healthcare to prepare nurses to understand the nature of global health
• Previous reviews on nursing students’ experiences with international clinical placement is limited to students from Western countries going to non-Western countries

Alt-text: Unlabelled box

**What does this paper add?**

• Students developed cultural competence through culture shock
• Cultural competence was learned not only during clinical practice, but also by living in a different culture
• Students experienced another way of practicing nursing due to the different nursing role, in which they mainly provided medical treatment and examination
• Supervisors were essential for students’ motivation and learning as well as making them feel safe

Alt-text: Unlabelled box

## 1 Background

Immigration, combined with the growth of multicultural societies, has increased patient diversity, highlighting the need for cultural competence in healthcare to prepare nurses to understand the nature of global health (Antón-Solanas et al., 2021; Pitkäjärvi, Eriksson, and Pitkälä, 2012; Turale, 2015). Cultural competence reflects the ability to provide effective, safe and quality care to patients while considering their cultural background and without infringing upon culturally sensitive issues (Antón-Solanas et al., 2021; Sharifi, Adib-Hajbaghery, and Najafi, 2019; Yu et al., 2021). Nurses require knowledge, abilities and skills to respect and understand how culture has an impact on individual and group health needs and on the decision-making process related to health promotion and restoration (Racine, Fowler-Kerry, and Palmer-Clarke, 2021; Yu et al., 2021). Without such competence, patients’ safety could be at risk in terms of delays in delivery of services or lack of adequate adherence to treatment, which could detrimentally impact patients’ health (Mkandawire-Valhmu and Doering, 2012). Papadopoulos (2018) developed a model consisting of three constructs that lead to cultural competence: cultural awareness, cultural knowledge and cultural sensitivity.

Knowledge of other cultures is an essential component of many undergraduate nursing programs across the globe (Antón-Solanas et al., 2021; Gradellini et al., 2021; Johnston, McKenna, Malik, and Reisenhofer, 2023; Yu et al., 2021). One strategy for achieving this is for educational institutions to offer international clinical placement, such as a semester exchange, a short-term study abroad program or a service-learning component where students travel to other countries to experience cultures different from their own (Johnston et al., 2023). International clinical placement can provide first-hand experience of the meaning of social determinants of health, the impact of the burden of disease and the effect of limited resources on global healthcare professionals. Such experiences can reduce the gap between theory and clinical practice (Cruz et al., 2017).

A systematic review explored factors that influence undergraduate healthcare students’ decision-making regarding study abroad opportunities (Brown, Boateng, and Evans, 2016). The review found wide variation in students’ motivation for international clinical placement, reporting that having sufficient information, encouraging interest in other cultures and countries and having academic staff as role models inspired students to study abroad. In another review exploring international clinical placement opportunities offered by undergraduate nursing programs in Australia, the findings suggested that development of cultural competence and increasing cultural awareness were important outcomes for students, who acknowledged the importance of having global awareness (Browne, Fetherston, and Medigovich, 2015). Another review summarised international nursing and midwifery students’ perceptions of challenge and enablement when undertaking an undergraduate program, finding that social isolation, language barriers, cultural barriers and adaptation to foreign teaching styles were challenging for nursing students (Eden, Fleet, and Cominos, 2021). The experience of socialization, unmet expectations and aspirations, and communication, cultural and relational challenges have also been reported to affect nursing students’ learning outcomes (Edgecombe, Jennings, and Bowden, 2013). Even though previous reviews have reported on students’ motivation and perceptions of studying abroad, to our knowledge, only one has focused on nursing students and was written within the last eight years.

Since international clinical placement is frequently applied as a learning activity in nursing education across the world, we conducted a scoping review including studies with different study designs and methods to summarise the range of research and the existing findings, as well as to identify gaps in the literature (Arksey and O'Malley, 2005). Such knowledge could be important to prepare nursing students, supervisors and teachers for international clinical placement and to increase nursing students’ learning outcomes from international clinical placement. Therefore, the aim of this scoping review was to provide an overview of published studies on undergraduate nursing students’ experiences during international clinical placement. The following research question guided our review: What is known from existing studies about undergraduate nursing students’ experiences during international clinical placement, and what research gaps exist?

## 2 Methods

### 2.1 Design

This scoping review used the five-stage methodological framework proposed by Arksey and O'Malley (2005), which consists of the following stages: identify the research question, study the selection, chart the data, collate the findings, and summarise and report the results. An appraisal of the methodological quality of the included studies was not performed, since this is usually not conducted in a scoping review. We did not conduct the optional stage of consultation, since three of the authors have extensive experience with international clinical placements. This collective experience was important to identify gaps in the existing studies. To optimise the quality of this review, the reporting of our findings followed the Preferred Reporting Items for Systematic Reviews and Meta-Analyses (PRISMA) Extension for Scoping Reviews (Tricco et al., 2018).

### 2.2 Search strategy

A systematic search was conducted in February 2023 using three databases: PubMed, Medline (Ovid) and CINAHL. The search strategy was built in PubMed (see Appendix 1) and adapted to the other databases. In addition, we conducted a hand search in the reference lists of included papers.

The identified publications were imported into EndNote to delete duplicates. Subsequently, the publications were imported into Rayyan software, which is a web-based tool designed to facilitate storage and blinding of the study selection process (Ouzzani, Hammady, Fedorowicz, and Elmagarmid, 2016). Two pairs of authors (SAS and LA; JS and BSS) independently screened titles and abstracts and then full-text papers for relevance based on the eligibility criteria. Discrepancies as to whether a publication should be included or not were deliberated, and consensus was reached through discussion among the pairs of authors.

### 2.3 Charting the data

A standardised data charting form was developed to extract relevant data from the included papers. The form included the following data: authors, year, country, aim, research design and method, sample size, country of clinical placement, length of clinical placement and findings related to the aim of the study. The same pairs of authors charted the data. One author extracted data, while the other checked the accuracy against the papers.

### 2.4 Collating, summarising and reporting the results

A scoping review aims to provide a map and summary of the findings; therefore, the analysis is descriptive (Pollock et al., 2021). In line with the framework of Arksey and O'Malley (2005), we used a qualitative approach to thematically group the data from the results section of the included papers using a low level of abstraction and an interpretation that closely adhered to the text. The data were read several times to obtain a thorough overview. Guided by our research questions, we identified patterns across the data from the included papers, which were then grouped into three thematic groupings. The first (LA) and last (BSS) authors grouped the data, while all authors agreed on the final thematic groupings. Previous scoping reviews have used such an approach for thematic groupings (Bingen et al., 2024; Nes et al., 2021; Skedsmo et al., 2023; Steindal et al., 2020).

## 3 Results

### 3.1 Selection of sources of evidence

**Fig. 1:**
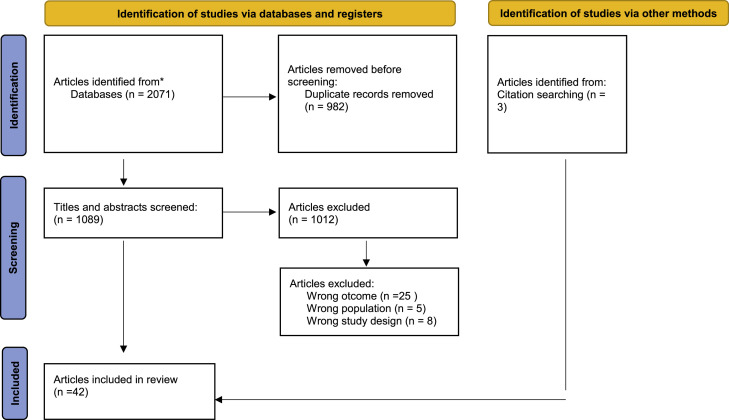
Preferred reporting item.

The systematic search yielded 2071 publications. After removal of duplicates (*n* = 982), 1089 titles and abstracts were screened. After reading the full-text papers, we included 39 studies, in addition to three papers identified through a hand search. A total of 42 studies were thus included in the review. The study selection process and the reasons for exclusion of full-text papers are shown in Fig. 1.

### 3.2 Study characteristics

**Fig. 2:**
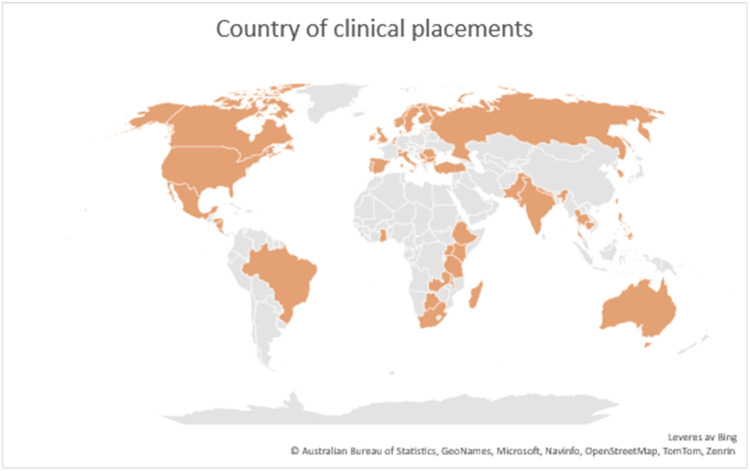
Country of clinical placements.

The included studies were published between 2008 and 2023 and 39 studies employed a qualitative design, while three studies employed a quantitative design (Bønløkke, van der Linde, and De Lorenzo Urien, 2018; Shelley Gower, Duggan, Dantas, and Boldy, 2019; Mikkonen, Elo, Miettunen, Saarikoski, and Kääriäinen, 2017). While the international clinical placement students came from many different countries, the majority were from Europe (Table 2). Further, most of the students had clinical placement outside Europe, and no studies included non-Western students with international clinical placement in a Western country (Fig. 2). While most clinical placements were in hospitals, some took place in community health centres or home-based care. There was a wide range of length of exchange, from two weeks to six months, and the age of the nursing students ranged from 19 to 50 years (Table 2).

### 3.3 Thematic groupings

Four thematic groupings were identified from the included studies: 1) developing cultural competence through culture shock, 2) a novel way of practicing nursing due to a different nursing role, 3) the essential role of the supervisor in learning outcomes and 4) the impact of language barriers on learning outcomes.

#### 3.3.1 Developing cultural competence through culture shock

Assimilating into a different culture cannot be learned from a textbook (Johnston et al., 2023). Many of the studies described cultural competence as a learning outcome from the international clinical placement

(Bønløkke et al., 2018; Gosse and Katic-Duffy, 2020; Shelley Gower et al., 2019; Halcomb, Antoniou, Middleton, and Mackay, 2018; Racine et al., 2021). Cultural competence included aspects such as being able to recognise their own culture, which enabled the students to view other cultures and determine how culture influences their behaviour and attitude (Peel, Missen, and Florentine, 2021).

Students experienced culture shock by being exposed to an unfamiliar environment, which allowed them to compare ways of performing practical nursing (Caldwell and Purtzer, 2015; Chaponniere and Hall, 2020; Ulvund and Mordal, 2017). They were overwhelmed by the poverty, as many patients could not afford to pay for their treatment or medication (Caldwell and Purtzer, 2015; S. Gower, Duggan, Dantas, and Boldy, 2017; Johannessen, Hovland, and Steen, 2014; Ulvund, Dadi, and Sundal, 2023). The situation made a special impact on those working with children, particularly when they observed children not given pain medication or antibiotics because their parents could not afford it (Sundal and Ulvund, 2023). Students were exposed to the deaths of newborns and to patients being admitted with various medical conditions who had experienced abuse, accidents, infections, cancer or burns. They even saw people lose their lives to illnesses that are preventable and curable in Western countries (Hovland and Johannessen, 2015). International clinical placement students noted different degrees of resource availability, manifested in the reuse of disposable items, violations of hygiene principles and a lack of warning tools to identify acutely ill patients in wards. This sometimes led to a sense of being overwhelmed and a feeling of helplessness (Chaponniere and Hall, 2020; Peel et al., 2021; Tjoflåt, Razaonandrianina, Karlsen, and Hansen, 2017; Ulvund et al., 2023). Students also experienced a huge gap in both quality and resources between public and private healthcare institutions (Johannessen et al., 2014).

Despite the reported culture shock, students described gaining increased competence with regard to different cultural values (Chaponniere and Hall, 2020; Gosse and Katic-Duffy, 2020; Levine, 2009; Peel et al., 2021) and a broader understanding of how difficult life may be for minority ethnic groups such as immigrants (Carpenter and Garcia, 2012; Green, Johansson, Rosser, Tengnah, and Segrott, 2008; Ulvund et al., 2023). The increased cultural competence and cultural awareness was reported to make students more understanding and accepting of cultural differences, especially in healthcare, enabling them to assess situations differently based on an alternative mind-set (Bohman and Borglin, 2014; Chaponniere and Hall, 2020). Further, expanding students’ horizons by engaging in self-reflection created an enhanced recognition of cultural differences and provided opportunities for transformative growth both personally and professionally (Gosse and Katic-Duffy, 2020).

International clinical placement participants compared healthcare systems, cultures and the availability of resources in their host and home countries (Hovland and Johannessen, 2015; Racine et al., 2021; Reid-Searl, Dwyer, Moxham, Happell, and Sander, 2011). Initially they focused on the disparities between the two healthcare systems (Huffman et al., 2020), but as time passed, recognition of similarities and respect emerged (Gosse and Katic-Duffy, 2020), which served as another way to increase their cultural competence (Ulvund and Mordal, 2017). Students had a definite slant towards identifying the strengths and weaknesses of the system in their own country (Keogh and Russel-Roberts, 2009). However, the contrast between nursing care in their home country and host country (Charles, 2015; Ulvund et al., 2023), the people they encountered and their own privileged lives left them struggling with feelings of guilt and discomfort (Caldwell and Purtzer, 2015). Recognising their own prejudices was a painful process for students (Levine, 2009), but they adjusted their prejudices and biases as time passed (Hagen, Munkhondya, and Myhre, 2009).

Exposure to different lifestyles, nursing practices and ways of problem-solving during international clinical placement enhanced students’ questioning and critical reflections about their work, their resource management and their approach to treating patients in their home country (Carter et al., 2019; Levine, 2009; Nielsen, Tarimo, Kibusi, Jansen, and Mkoka, 2020; Peel et al., 2021), and these critical reflections were reported to augment students’ learning (Afriyie, Reimer-Kirkham, and Astle, 2013).

Students learned cultural competence not only during the clinical practice, but also by living in a different culture. Some students experienced homesickness and concerns that revolved around the external environment, crime and disorder. They were uncertain about how they should behave and what was safe or unsafe (Ulvund and Mordal, 2017), and some experienced the society or the political situation as unsafe (Green et al., 2008). Students reported problems sleeping due to noise, hard mattresses, heat and humidity, dirty rooms and toilet facilities, and water and electricity restrictions, while others had food problems leading to either constipation or diarrhoea (Chaponniere and Hall, 2020; Charles, 2015). Some students felt like they were living under a microscope and often felt stared at wherever they went (Peel et al., 2021), or that they were verbally harassed by the locals (Chaponniere and Hall, 2020). Other students reported the challenge of not being able to go out at night without being accompanied by a man, something that was difficult for some female students who were used to being independent (Maltby, de Vries-Erich, and Lund, 2016). Nevertheless, students acknowledged learning from the feeling of being a minority in the host country (Gosse and Katic-Duffy, 2020).

8 out of 27 studies in this thematic group reported using a theory or model. Three studies used the Campinha-Bacote's theoretical framework process of cultural competence in the delivery of health care model (Shelley Gower et al., 2019; S. Gower et al., 2017; Hagen et al., 2009), one study applied a postcolonial feminist theoretical approach (Racine et al., 2021), one study Leininger's Theory of Cultural Care (Peel et al., 2021), one study used Papadopoulus, Tilki and Taylor's Model for Developing Cultural Competence (Ulvund and Mordal, 2017). Furthermore, one study used Oberg’s (1960) original model (4-stage adjustment) (Chaponniere and Hall, 2020) and one study used a cultural competency continuum to describe the process of becoming culturally competent (Maltby et al., 2016).

#### 3.3.2 A novel way of practicing nursing due to a different nursing role

Students recognised differences regarding the nursing role in their host and home countries and found that nurses often provided care to suit the cultural context and the available resources (Afriyie et al., 2013; Carter et al., 2019; Gosse and Katic-Duffy, 2020; Torsvik and Hedlund, 2008). According to students, nurses in non-Western countries mainly performed medical treatment and examinations, diagnosed fractures using x-rays, corrected bone positions, gave medications and nutrition and, to a lesser extent, provided hands-on care (Sundal and Ulvund, 2023). For some students, these differences in the nursing role created discomfort and the experience of an unclear nursing role (S. Gower et al., 2017)Students reported that nurses memorised knowledge, treated symptoms without investigating causes and diagnosed without using tests (Gosse and Katic-Duffy, 2020). Nevertheless, students were given opportunities to use their practical skills, performing procedures they did not do in their home country, such as applying stitches, giving injections, participating in deliveries (Johannessen et al., 2014) and treating malnourished children (Ulvund et al., 2023). They also learned about infectious and tropical diseases (Bohman and Borglin, 2014; Maltby et al., 2016) and how to perform nursing procedures with fewer resources. In addition, they became more aware of what kind of equipment to employ for different procedures, since patients paid for the equipment used (Hovland and Johannessen, 2015; Peel et al., 2021).

In students’ view, nurses in the host countries lacked focus on mental health (Grudt and Hadders, 2018) and did not focus on basic nursing care, such as ensuring that patients ate sufficiently or were given a bath when lying in urine and faeces (Johannessen et al., 2014). Furthermore, students felt that the nurses had a limited connection with patients, lacked empathy and respect when patients cried, and treated dying or dead patients in an undignified manner. They sometimes found that nurses lied to patients, laughed at them and shared patient information without a clinical purpose. Furthermore, nurses in the host countries became angry when patients did not do what was expected, and in some places they punished patients physically or verbally for expressing pain (Bagnasco et al., 2020; Grudt and Hadders, 2018; Johannessen et al., 2014; Levine, 2009).

Performing nursing in a different context also made students aware of the power that nurses have over patients. Even if students were aware of this power in their home countries, it became more obvious when they performed nursing in a different context (Hagen et al., 2009). However, students were concerned about doing more harm than good, and they questioned whether their presence and actions could impact vulnerable communities, causing the students to consider the ethical dilemma of malfeasance (Caldwell and Purtzer, 2015; Jørgensen and Hadders, 2015; Morgan, 2019; Tjoflåt et al., 2017). Students felt insecure as they faced a conflict between their own professional values and the instructions given by local staff (Hagen et al., 2009; Ulvund and Mordal, 2017).

Encountering different ways of performing nursing, e.g. families taking care of patients’ basic needs (Carter et al., 2019; Charles, 2015; Ulvund and Mordal, 2017), as well as differences in values and respect (Bønløkke et al., 2018), challenged students to think and expand their knowledge (Bagnasco et al., 2020). They became more aware of patient rights from incidents and discussions with staff (Green et al., 2008; Hagen et al., 2009) and reflected on social injustice based on what they witnessed (Afriyie et al., 2013). Furthermore, students recognised how fortunate they were to live in their home country, with economic resources and well-functioning institutions, and they came to appreciate the healthcare offered there (Carter et al., 2019). To process their international clinical placement experiences, students agreed about the need for time to reflect on their experiences (Afriyie et al., 2013; S. Gower et al., 2017; Hovland and Johannessen, 2015; Jørgensen and Hadders, 2015; Ulvund et al., 2023). They used reflection logs and journals to improve care through an understanding of the people and referred to the importance of systematic thinking in their clinical experiences to evaluate past choices and to identify learning outcomes (O'Donnell et al., 2022). Having at least two students together during international clinical placement was beneficial, as they could discuss and share their experiences while supporting each other (Reid-Searl et al., 2011; Ulvund et al., 2023), thus enabling them to improve their knowledge (Jørgensen and Hadders, 2015; Peel et al., 2021; Tjoflåt et al., 2017).

#### 3.3.3 The essential role of the supervisor in learning outcomes

Supervisors from the host organisation were described as being in an essential position to encourage and shape students’ learning (Afriyie et al., 2013). These supervisors were important for students’ motivation and inspiration, influencing their ability to attain knowledge and playing a crucial role in students’ success (Afriyie et al., 2013; Bagnasco et al., 2020; Mikkonen et al., 2017; Siles Gonzalez, Solano Ruiz, and Gaban Gutierrez, 2016). While some students described their supervisor as a qualified reflection partner, helping them consider the experiences in the context they encountered (Ulvund et al., 2023), other students reported that the supervisors protected them from discriminatory behaviour (Masera, Ngo Bikatal, Sarli, and Sarli, 2015). This made students feel safe, provided them with emotional support and helped them develop an open mind and cultural competence through guided reflection (Ulvund and Mordal, 2017).

In contrast, some students felt that the supervisors were impatient, which caused them stress (Masera et al., 2015). Sometimes supervisors were perceived as reluctant to include students in the clinic, while others were inclusive and took time to answer questions, even when they were quite busy (Jørgensen and Hadders, 2015). Altogether, the clinical environment and supervision in a clinical setting enabled students to practice skills under the supervision of mentors, which played an essential part in their development as nurses (Mikkonen et al., 2017).

#### 3.3.4 The impact of language barriers on learning outcomes

Language barriers were a significant challenge for students, and they did not understand how they would overcome the language barrier before they met patients who did not speak their languages (Halcomb et al., 2018; Myhre, 2011). Even being able to communicate in English was a source of anxiety and distress because of different dialects and pronunciations, which impacted students’ communication and relationship with patients, relatives, supervisors and staff (Bohman and Borglin, 2014; Grudt and Hadders, 2018; Jørgensen and Hadders, 2015; Siles Gonzalez et al., 2016). Therefore, having some native language capacity to communicate with patients and staff was considered a critical skill (Racine et al., 2021).

Language barriers between supervisors and students also posed a significant challenge in achieving learning outcomes (Carpenter and Garcia, 2012; Masera et al., 2015; Mikkonen et al., 2017). Despite supervisors being willing to help, language difficulties made students afraid to explain their problems, as they did not know how to express themselves (Masera et al., 2015; Myhre, 2011; Ulvund and Mordal, 2017). Nevertheless, language barriers increased some students’ learning outcomes as they were forced to use nonverbal skills (Ulvund and Mordal, 2017), through which they were better able to communicate (Grudt and Hadders, 2018; Huffman et al., 2020; Jørgensen and Hadders, 2015; Racine et al., 2021). Students with stronger language skills had a better outcome related to cultural competence in international clinical placement than those at the beginner level (Racine et al., 2021). However, newly learned language skills and knowledge about other cultures also helped students to deliver better patient care (Caldwell and Purtzer, 2015; Carpenter and Garcia, 2012) and to be aware of what their future patients might experience (Peel et al., 2021).

## 4 Discussion

This scoping review aimed to provide an overview of published studies on undergraduate nursing students’ experiences during international clinical placement.

According to Leininger (2002), cultural competence and understanding is important and can help nurses provide quality nursing care. However, research indicates that cultural competence is lacking among nurses (Alpers and Hanssen, 2014; Shahzad, Ali, Younas, and Tayaben, 2021). International clinical placement may provide students diverse learning opportunities to cultivate and achieve outcomes related to global nursing, expanded perspectives to explore and perform in a new climate, self-efficacy and critical thinking skills (Browne et al., 2015; Cruz et al., 2017; Edmonds, 2012). Such experiences can enable nurses to provide safe and quality care while considering the patient’s cultural background (Antón-Solanas et al., 2021; Yu et al., 2021). Our findings suggest that international clinical placement students may develop cultural competence through experiencing the culture shock of being exposed to an unfamiliar cultural environment, such as different ways of addressing patients’ pain or carrying out procedures and hygiene—even when students may initially react negatively to these differences. This is in line with previous research where supervisors reported that nursing students who were shocked about the way procedures were carried out and who struggled to understand how to meet human needs with fewer resources gained new insight, as their initial emotions and reactions sometimes changed when they were given an explanation (Strøm, Sarin, Steindal, and Andreassen, 2023). Being surrounded by poverty (Caldwell and Purtzer, 2015), different perceptions of cleanliness and a shortage of material resources may be troublesome for students (Kokko, 2011). However, our review suggests that students tend to adjust their prejudices and biases as time passes and as they discover the cultural values, experience enhanced understanding and accept cultural differences. Nevertheless, to gain such insight and understanding, the length of the international clinical placement might be an important factor. Some argue that periods shorter than four weeks may hinder students' ability to fully engage with the host culture. Conversely, others contend that extended placements may not be necessary to foster a shift in students' attitudes from ethnocentrism toward the initial development of culture sensitivity (Browne and Fetherston, 2018). Consequently, future research should focus on identifying and recommending the ideal duration for international clinical placement.

Our review determined that students experience another way of practicing nursing due to a different nursing role, one mainly responsible for providing medical treatment and examination. Nurses in non-Western countries were observed to memorise knowledge rather than focus on critical thinking and processing, which is the predominant Western method (Ramburuth and McCormick, 2001). This finding is consistent with a where nursing students reported that the nursing role was different from what they were used to in their home country study (Meyer, Søvik, and Strøm, 2025). Nevertheless, Strøm et al. (2023) emphasised the need to balance the Eastern and Western approaches. The Eastern approach emphasises a Confucian method of learning where students follow the supervisor’s instructions, believe what the supervisor says is true and memorise what is said. To the contrary, the Western Socratic approach encourages students to think for themselves (Campbell and Li, 2008). As an example of how these two approaches might be blended together, demonstrations by supervisors may be needed to learn nursing procedures (Eastern approach), whereas interaction with patients and family can be better learned through reflection (Western approach) (Strøm et al., 2023).

We found that students experienced both violations of professional values and ethical encounters during international clinical placement. These experiences may also be related to students’ experiences of another way of practicing nursing. A systematic literature review found that nursing and medical students often encounter ethically complex situations during international clinical placements, particularly when patients were not treated with respect (Rahim, Knights, Fyfe, Alagarajah, and Baraitser, 2016). Addressing such situations requires respect for the host community's values, beliefs, and customs, rather than operating from an assumption of high-income country superiority. Visiting students must prioritize relationship-building and dialogue to avoid perceiving or treating others as less than a full person (Yoder, Soule, Nguyen, and Saluta, 2022). To navigate these ethical complexities, students need to cultivate skills such as critical reflection, cultural competence, and a willingness to listen and learn (Rahim et al., 2016). Ethical frameworks, such as the 4 biomedical principles (autonomy, beneficence, nonmaleficence, and justice) or alternative models specifically tailored for international clinical placements in low- and middle-income countries, may guide decision-making in such situations. These alternative models recognize cultural variations in interpreting concepts such as autonomy, the dual role of the student as both a visitor disadvantaged by limited knowledge of the local language and culture and as someone advantaged by greater access to resources and the inherent tension between cultural pluralism and universal human rights (Rahim et al., 2016). Future studies should involve students, teachers and supervisors in the co-design and evaluation of interventions to support nursing students and supervisors in effectively managing ethically complex situations during international clinical placement.

By ensuring adequate preparation, fostering cultural competence, maintaining open communication and monitoring clinical competence, supervisors have been reported to contribute significantly to the success and positive impact of nursing students engaged in practicing abroad (Kristofferzon, Mårtensson, Mamhidir, and Löfmark, 2013). This concurs with our findings, in which students described supervisors as essential for their motivation and learning as well as for making students feel safe during international clinical placement. When supervisors were asked how they view their role when supervising students from Western countries, they emphasized the importance of protecting them (Strøm et al., 2023).

Edgecombe et al. (2013) suggested that socialization, communication, relationships, unmet expectations and aspirations affect students’ learning during international clinical placement. Having a successful learning experience with effective communication is crucial in nursing, especially when interacting with patients. However, in line with previous research (Kelleher, FitzGerald, and Hegarty, 2016; Kumwenda, Royan, Ringsell, and Dowell, 2014; Unver, Cakiroglu, and Satilmis, 2021), our review found that language skills were considered one of the greatest barriers for international clinical placement students. The same challenge was experienced by supervisors, who expressed greater frustration regarding language barriers than cultural barriers (Newton, Pront, and Giles, 2018). Nursing has its own complex and specialised vocabulary. Learning nursing terminology in a foreign language adds an extra layer of complexity for students. Language barriers could make it challenging for students to establish a therapeutic relationship with patients and their relatives (Jansen et al., 2021). Furthermore, language barriers may impede students’ ability to gather accurate medical histories, explain procedures, provide emotional support and collaborate with other healthcare professionals. In addition, language barriers may present challenges regarding provision of high-quality healthcare and patient safety (Al Shamsi, Almutairi, Al Mashrafi, and Al Kalbani, 2020). However, our review also suggests that language barriers could enhance students’ learning outcomes, as they forced students to improve their language skills, both verbally and nonverbally, and gain knowledge about other cultures.

All the studies in our review described Western students with international clinical placement in a non-Western country. Although studies on this population provide valuable insights, there is a need to investigate how non-Western students experience studying in a Western country and to elaborate on any differences in that experience. A limited number of the included studies used a theory or model concerning development of cultural competence. Since students' growth in cultural competence seems to be crucial to the success of international clinical placement, future studies should use a theory or model to understand how students develop cultural competence and to develop interventions to increase such competence. Furthermore, almost all the studies included in our review used a qualitative design (*n* = 39). These are limited by the fact that broad generalizations cannot be suggested, as the findings are from the experience of the study sample only, rather than the population at large. Future studies should employ quantitative designs to examine students’ learning outcomes such as cultural competence.

### 4.1 Limitations

Firstly, there may be search terms regarding international clinical placement that were not included in the search strategy. Only three databases were searched; however, these databases index studies within nursing education. In line with the aim to provide an overview of published studies, grey literature was not included. It is the aim of the review that decides whether grey literature should be included in a scoping review (Tricco et al., 2018). Secondly, most of the included papers reported students from Western countries and their experience of conducting international clinical placement outside of Europe. However, such experiences could vary greatly among students from different countries. Thirdly, students’ international clinical placement ranged from two weeks to six months, which meant that they had diverse experiences, and their international clinical placements were mostly conducted in hospitals. This could limit the transferability of the findings. Finally, the findings must be interpreted with caution since the methodological quality of the included studies was not appraised and the data were descriptively analysed, in line with scoping review methodology (Arksey and O'Malley, 2005).

## 5 Conclusion

Students developed cultural competence through experiencing culture shock when they were exposed to an unfamiliar cultural environment. While they often reacted negatively to these differences at first, they seemed to adjust their prejudices and biases after some time had elapsed. Language barriers were reported to be the greatest challenge, creating more frustration than cultural barriers. There is a need to investigate how non-Western students experience studying in a Western country. Tables 1 and 3

**Table 1:** Origin of students.

Origin of students	No
Norway	13
US	7
UK	6
Australia	6
Canada	3
Denmark	2
Finland	1
Ireland	1
Italy	1
Sweden	1
Germany	1
Japan	1
Spain	1
Madagascar	1
Malawi	1

**Table 2:** Eligibility criteria.

Criterion	Inclusion	Exclusion
**Type of study**	Quantitative, qualitative or mixed methods study on the phenomenon, published in peer-reviewed journals	Master’s theses, doctoral theses, reviews, conference abstracts, conference proceedings, editorials, comments, letters, reports, guidelines, books or book chapters
**Participants**	Undergraduate nurses who participated in international clinical placement	Postgraduate nursing students or other healthcare profession students
**Phenomenon of interest**	Undergraduate nursing students’ experiences of international clinical placement	Pre-departure preparations, observational studies or learning services
**Period**	1 January 2008 through February 2023	
**Language**	English, since all authors understand this language

**Table 3:** Characteristics of the includes studies.

Authors, year, country	Aim	Research design	Sample characteristics	Country of clinical placement	Context	Length of clinical placement	Key findings
Afriyie et al. (2013), Canada	To explore how nursing students (NSs) learn during the international experience	Qualitative design	*N* = 8; 8 females, mean age not reported (NR), age range 19–24 years	Zambia	Hospital, outpatient clinic, traveling health unit	3 weeks	NSs had seen repeated images of Africa as a continent of poverty and great need which contrasted with the modern city that greeted them. A strong system of support was nurtured through learning in pairs. Journal writing prompted NSs to reflect on situations that fostered learning. The care provided was different from what they knew. Zambian nursing knowledge was similar to their own; they just practiced in a different way to suit the cultural context and the available resources
Bagnasco et al. (2020), Italy	To understand the impact of participation to an international clinical placement experience on undergraduate NSs educational learning, and to determine factors facilitated the NSs learning outcomes during the exchange	Descriptive qualitative design	*N* = 6; 4 females, mean age 23.5 years	Italy, Portugal, Spain	University sites	Median of 5 months	NSs choice of exchange institution was linked to their knowledge or motivation to the city available for exchange. They had thoughts and feelings ranging from fear and disorientation for being alone, a new culture or the language, to excitement about a new experience. Learning outcomes that permitted NSs to grow and address changes in personal, improved professional awareness, and extended their viewpoint through contact with a different work environment
Bohman & Borglin (2014) Sweden	To explore Swedish NSs’ perceptions and experience of student exchange	Descriptive qualitative design	*N* = 9; 6 females, mean age 27 years, age range 19–32 years	Nordic countries, European Union (EU), non-EU	Different healthcare institutions	2–12 weeks	Reasons for applying for exchange programme were desire to have new knowledge of different technical equipment, facilities, structural organisation, and hierarchies. Student exchange was viewed as an opportunity to experience cultural insight and reflected on “being prepared” as a registered nurse (RN) for the multicultural society. Student challenges were language barriers and exposure to different education system resulting in a feeling of personal growth and maturity in terms of being more self-confident and self-aware
Bønløkke et al. (2018), Denmark	To enable nursing students to benefit from a short-term exchange, offering the opportunity to experience different societies, their culture and nursing culture	Qualitative design	*N* = 329; 91 % females, mean age NR, 6 % aged under 20 years, 77 % 20–25 years, 10 % 25–30 years and 7 % over 30 years	Belgium, Bulgaria, Denmark, Italy, the Republic of Macedonia, the Netherlands, Norway, Romania, the Russian Federation, Serbia, Spain, Sweden, Switzerland, the UK	29 different nursing institutions	2 weeks	NSs valued time to observe and understand, awareness of their own culture, a positive attitude, opportunities to compare differences and similarities and sharing and communicating. These situations helping them to acquired professional cultural learning and mental processes towards understanding cultural issues
Caldwell & Purtzer (2015) US	To discover long-term learning outcomes in a short-term study abroad programme	Descriptive qualitative design	*N* = 41; 39 females, mean age NR, age range 22–60 years	Honduras	Community healthcare service	10–12 days	The realization that one can live in poverty and still be happy, feel fulfilled, and be consent appeared frequently in the responses. NSs felt guilty on the stark contrast between the people they encountered and their own privileged lives. Some had placed increased importance on self-reflection after returning home. A strong concern for doing more harm than good emerged as NSs questioned how their presence could impact a vulnerable community
Carpenter et al. (2012), US	To find out how study abroad experience influenced the participants' awareness, sensitivity, knowledge, and skills, and how it influenced participants clinical placement as nurses	Descriptive qualitative design	*N* = 35; 85,7 % females, mean age NR, age range 9–35 years	Mexico	Community healthcare institutions	4–6 weeks	A change in cultural awareness and understanding of the hosts culture, cultural sensitivity with growing respect for cultural differences, cultural knowledge to understand the differences between own culture and the hosts culture, and cultural skills with improved language skills. More cultural knowledge and better language skills would help deliver better patient care
Carter et al. (2019),UK	To evaluate the impact of a faculty-structured international travel elective to Zambia for undergraduate UK nursing students	Descriptive phenomenology	*N* = 6; gender NR, age NR	Zambia	NR	NR	NSs reported the value of the pre-elective training pathway preparing them for their elective, but this in addition to their normal studies were tiring. NSs were concerned about reuse of disposable equipment, lack of early warning scoring tools to identify the acutely ill patient and lack of access to basic equipment. The hierarchical nature of the Zambian healthcare system was challenging. Due to lack of RNs, students had to prioritize patient care which resulted in few opportunities for bedside teaching or mentorship. The importance of resource management could change their practice when they returned to UK
Chaponniere et al. (2020), US	To identify major themes in student journals entries as they describe their cross-cultural experiences	Descriptive qualitative design	*N* = 55 senior NS; 52 females, mean age NR, age range 22–50 years	Ghana	Community health service	2 weeks	Positive coping skills included need for control, humour, normalizing, reframing, need for breaks and relaxation. Venting, frustration, and physical ailments were coded as negative responses to culture shock
Charles et al. (2015), Australia	To describe the immersion experience of a group of senior Australian nursing students who participated in a 5-week cultural immersion programme in India	Qualitative descriptive	*N* = 8; gender NR, age NR	India	Community clinics, hospitals	5 weeks	NSs were struck by the differences between Australian and Indian healthcare provision and systems. NSs thought of their way as the ‘right’ way to do things. After NS had spent time with the nurses in CP they to appreciate the differences in healthcare provision, and to see and understand how healthcare in India worked
Dohrn et al. (2018), US	To learn how RNs in host health facilities acclimate to challenges like the burden of disease and limited resources	Qualitative design	*N* = 24; gender NR, age NR	Malawi	5 different healthcare institutions	6 weeks	Clinical exchange provides a transformative addition to nursing education with a deepened understanding of health disparities and nursing roles in different health systems, sharing experiences and knowledge
Gosse et al. (2020), Canada	Visiting and host students and faculty’s perceptions of reciprocity during international learning experiences	Descriptive qualitative design	*N* = 10; gender NR, age NR	Jamaica	Regional hospital	2 weeks	Mutual benefits to host and students during clinical learning were reflected through; mutual gains were noted between host and visiting NSs and faculty; similarities outweighed the differences between healthcare and culture; personal and professional growth occurred for participating students and faculty; knowledge sharing and intercultural collaboration developed mutual cultural awareness
Gower et al. (2019) Australia	To explore the influence of international clinical placement experience on cultural competence of participants in short term and 12 months later	Longitudinal study	*N* = 50; 47 females; mean age 27.1, range 19–55	Cambodia India, Philippines, Thailand, Tanzania	Rural community clinics and communities, public and private metropolitan hospitals	Between 2 and 4 weeks	Significant change in Mean total scores from time 1 to time 2 showed that participants moved from being culturally aware to culturally competent. No significant change in mean total score between time 2 and time 3. Significant increase in total scores occurred following the placement which were maintained for 12 months
Gower et al. (2017) Australia	To examine the understandings of global health issues among nursing students following participation in an international clinical placement	Descriptive qualitative design	*N* = 25; gender NR, age NR	Tanzania, Thailand, The Philippines, Cambodia, India	Public and privately operated hospitals	3 months	For the majority of pa. rti The experience of a healthcare system that required payment from the patients, made NSs reflect over their own healthcare system taken for granted. They felt overwhelmed and found the facilities and resulting impacts on patients difficult to accept. Differences in sterility, documentation, dispensing of medication and general hygiene were confronting. NSs expressed a broader view of the world and a greater passion for discussing international issues. NSs found it difficult adapting to personal and professional life back at home
Green et al. (2008), Sweden and UK	To examine the experiences of nursing students undertaking an international placement during their pre-registration education	Multi case study	*N* = 32 (18 from UK, 14 from Sweden), 28 females, mean age NR, age range 20–49 years	Sweden, Holland, Spain, Denmark, Finland, Wales, USA, Pakistan, South Africa	NR	Swedish students at least 10 weeks UK students’ number of weeks NR	NSs described an increase in confidence, self-reliance and professional knowledge and skills resulting from their international placement. There was an awareness of how healthcare roles differ between countries and a change in attitudes to others from different backgrounds and cultures. The was a marginal difference between the two cases. The support from home and host universities varied between the international placement providers
Grudt et al. (2018), Norway	To gain understanding of Norwegian student’s' practical experience of culture sensitivity	Qualitative design	*N* = 7; gender NR, age NR	Nicaragua	Different health institutions and local hospitals	10 weeks	Positive findings: Increased awareness about the nursing discourses and power relations shaping clinical encounters throughout their learning trajectory in CP. Increased awareness of the politics of nursing practices through their experiences of clashes between different nursing discourses Negative findings: Breach of confidentiality, challenge of ethical values, communication challenges with their colleagues, the patient and next in kin, different nursing role and work environment
Hagen et al. (2009), Norway	To reveal and describe the experiences that Norwegian and Malawian nursing students had on shared clinical placement in Malawi in 2006	Phenomenological approach	*N* = 5 (3 Norwegian students, 2 Malawi students); gender NR, age NR	Malawi	Hospital and community care	8 weeks	NSs experienced similarities and differences in practice, but similarities are regarded as the stronger impression. Learning relational skills was the primary learning outcome, but learning how to nurse patients was also an important outcome. All NSs developed cultural competence during CP
Halcomb et al. (2018), Australia	To explore the experiences of Australian undergraduate nursing students undertaking a primary healthcare CP in Cambodia	Exploratory qualitative	*N* = 8; 7 females, mean age 21.6 (standard deviation (SD) 3.4) years	Cambodia	Primary health clinics	2 weeks	Despite pre-placement preparation, NSs were challenged during the CP in ways that they never expected which made them grow professionally and personally. Exposing NSs to primary healthcare in practice helped to demonstrate the value and importance of this area of nursing
Hovland & Johannessen (2015), Norway	To identify the features that characterized students’ experiences reported in their reflective journals during clinical placement in an African country in light of cultural competence	Qualitative design	*N* = 197; gender NR, age NR	Botswana, South-Africa or Tanzania	Hospitals, healthcare centres, home-based care	8–12 weeks	Reflective journals were characterized by the students’ personal emotions, judgements of others and comparisons between what they had learned in Norway and what they experienced in the African country in which they interned
Hovland & Johannessen (2018), Norway	To gain an insight into nursing students personal experiences with developing cultural competence during a student exchange in Tanzania	Qualitative design	*N* = 21; 19 females, age NR	Tanzania	Health clinics, day centres, school health services and retirement homes, and in mental health services in home nursing and outpatient clinics	3 months	Development of cultural competence was dependent on NSs being given explanations of aspects they did not understand and having their attitudes challenged. They developed an increased understanding by participating in cultural encounters with people over time and by reflecting on their personal experiences. Maintaining an open attitude in their cultural encounters with people and situations combined with a willingness to go outside their comfort zone also helped the students to develop cultural competence
Huffman et al. (2020), Japan	To qualitatively analyse the experiences and perceptions of students at a nursing college in Japan who studied abroad in Asia and North America, thereby identifying the full range of benefits of study abroad programmes for Japanese NSs	Qualitative design	*N* = 50; gender NR, age NR	US, Philippines, Thailand, South-Korea, Taiwan or Canada	NR	10 days-8 weeks	Perceived benefits in English language proficiency and motivation were knowledge of nursing practices, healthcare systems, and global health; cultural awareness and sensitivity; and various types of identity development (second-language motivation and identity, national/ethnic identity, professional identity, identity as a global citizen, and personal growth). NSs’ perceptions of what they learned or gained varied according to the specific characteristics of each study abroad programme
Jansen et al. (2020), Denmark, England, and Ireland	To capture the experiences of cultural, personal and professional development during international clinical placement among nursing students from 3 European countries	Qualitative design	*N* = 23 (10 short international CP, 13 long international CP); 23 females, mean age NR, age range 21–47 years	Cypres, US, UK, Turkey, Kenya, Finland, Ireland, Norway, Thailand, Tanzania, Denmark	Hospitals	Short international CP 3–4 weeks Long international CP 10–12 weeks	The international CP impacted on the NSs personal and professional way of understanding themselves as students and future RNs. A profound difference was seen between the achieved learning outcomes of NSs completing an international CP in a high- or low–income country. Language barriers, local culture and different nursing cultures were often challenging and pushed NSs out of their comfort zone. All NSs developed their cultural understanding
Johannessen et al. (2014), Norway	To gain knowledge of the topics these students were concerned with during their placement by analysing their journals	Qualitative design	*N* = 197, gender NR, age NR	Botswana, South-Africa or Tanzania	Hospitals, healthcare centres, home-based care	12 weeks	NSs perceived negative attitudes such as lack of planning, structure, management and monitoring, unprofessional communication, and that nurses lacked knowledge among nurses. NSs sometimes experienced their role as important, perceived violation of hygiene principles, and the lack of personnel, equipment, electrical power, and money
Jørgensen et al. (2015), Norway	To gain understanding of Norwegian students’ experience of learning in clinical placement in Bangladesh without formal one-to-one supervision, by a personal mentor in the ward	Qualitative design	*N* = 7; gender NR, age NR	Bangladesh	Hospital	7 weeks	NSs felt excluded from performing “hands on” or expected to perform “hands on” CP without relevant preparation and guidance. Being a group was important to overcome overwhelming impressions and cultural encounters. Breaks were important to get control of the situation and to obtain more predictability in their daily life. By time NSs got sufficient clinical experience which enable them to feel safe enough to take initiative and perform procedures on their own. After CP NSs talked about achieving their learning objectives related to outcomes, nursing skills and nursing identity in a broader perspective
Keogh & Russel-Roberts (2009), Germany	To find some evidence about the success of this specific exchange programme by asking the students to identify their benefits from their experience, instead of merely looking at the numbers of students in the exchange programme	Qualitive approach	*N* = 7, gender NR, age NR	Finland	Hospital	Minimum 20 weeks	NSs compared the nursing competencies of nurses in both countries while being in the host-country. NSs wanted to change and/or challenge the German system. Some had difficulties in talking to patients, but fewer problems in communicating within the nursing team, as English or German was known to all the supervisors. All were interested in learning about new cultures. NSs wanted supervision, but some also wanted to be able to act self-directed when gathering own experiences. Their personal approach to the delivery of nursing care changed dramatically after CP
Levine (2009),US	To describe nursing students' viewpoints while participating in international immersion programmes	Qualitative inquisitive methods	*N* = 10: gender NR, age NR	Central America, Southern Asia, Eastern Europe	Hospital, community heath centre, people’s home, public health	6–9 weeks	NSs described blind trust as acceptance based on seeing things as they are and they described this by caring, accepting, being open, understanding, and being concerned. Transforming experiences, included taking risks, lead to personal growth such as increased awareness as they were challenged to move beyond their comfort zone. NSs discussed their own discriminatory beliefs and biased feelings they did not realize they had
Maltby (2016), US	To understand the experience of American nursing students who complete a study abroad trip to a low-income country, Bangladesh, versus a high-income country, the Netherlands in the development of cultural competence	Hermeneutic phenomenology	*N* = 45; 42 females, mean age 22 years	Bangladesh Netherlands	Public health nursing	3 weeks	The theme unique to the Bangladesh groups was they are poor/ I am wealthy, while the unique theme unique for the Netherlands group was the realization that primary care is the central focus of the healthcare system. There were two common themes for both groups: 1) public health; 2) stranger in a strange land. In Bangladesh IV were started within two minutes and was good if not better than any hospital in the US. NSs experienced a cultural shock where food and water tended to be the topic. Language barriers was another challenge, which caused frustration. They felt like a foreigner and started to empathize with immigrants
Masere et al. (2015), Spain	To explore the experience of students from Cameroon studying on the degree courses in Medicine and Surgery and in Nursing at the University of Parma, in order to discover the strong and weak points of the organisation of the course so as to achieve a good process of integration	Ethnographic study	*N* = 20 (from Cameroon); 5 females, mean age 26.5, age range 24–31 years	Italy	NR	1 year	NSs felt that the Italian NSs adopt detached, distant, and reserved behaviours towards them. Some claimed that they had been met with discriminatory and racist attitudes. Some mention that African NSs should put more effort into their relationships with others. NSs also had some positive experiences.7 The nursing staff were considered being kind and willing to help. NSs felt that it was difficult to speak to or approach teachers since they were used to a bigger distance between students and teachers in Cameroon. Some NSs criticised the Italian university system who seemed to be more focused on theorical rather than practical skills
Mikkonen (2017), Finland	To describe international and national students' perceptions of their clinical learning environment and supervision, and explain the related background factors	Explorative cross-sectional design	*N* = 231 (Africa (29.8 %, Europe 15.8 %, Asia14.9, North America 7.0 %, South America 0.3 %); 156 females, mean age 28 years	Finland	Primary healthcare and specialized medical care	2 weeks or more	International NSs at a beginner level in Finnish perceived the pedagogical atmosphere as worse than native speakers. These international students generally needed greater support from the nurse teacher at the university. NSs at an intermediate level in Finnish reported two times fewer negative encounters in cultural diversity at their CP than the beginners. NSs were generally satisfied with the content of supervisory relationship, including a positive attitude toward them, individual supervision, and mutual respect and trust by mentor
Morgan (2012), UK	To investigate UK undergraduate student nurse experiences of risk during an international placement	Phenomenological	*N* = 10, 10 females, mean age 20 years	EU countries, Non-EU countries, Developing countries	NR	Either 12 weeks Erasmus CP or short worldwide CP	NSs got scared when told that poor people who could not pay for their treatment would do anything to get the treatment. Language barriers was a challenge in the relation with patients. NSs felt that they did not fit in and was afraid to be at risk of social isolation. Being conscious about what they were doing were highlighted. They compared with what they were familiar with and made friends with people and decided that the strangers were friendly. The exposure to risk helped them to learn about them-self and to develop a cultural competence
Morgan (2019), UK	To gain insight into learning processes, strategies employed, and influences on learning throughout the study abroad journey	Interpretivist hermeneutic phenomenological methodology	*N* = 20; gender NR, age NR	EU countries, Developing countries	NR	1–3 months	The motivation to study abroad was the possibility to experience something completely different. NSs experienced disjuncture, culture shock and troublesomeness despite the anticipations they had beforehand. Being with people and the feeling of being accepted, played an influential role in learning. By studying abroad, NSs became different to how they were prior to the experience, felt more independent, confident and progressed towards cultural competence. It took time to adjust when they came home again
Myhre (2011), Norway	To examines challenges and learning outcomes for nursing students from a Central European university of applied sciences who completed 3 months of clinical placement in Norway	Explorative and descriptive with a hermeneutical approach	*N* = 3 (from Central Europe); 2 females, mean age NR, age range 21–25 years	Norway	Hospital	3 months	Greater self-confidence was the most important outcome of the exchange as they had to find out things on their own. NSs were trained to use non-verbal communication techniques and became more structured and independent in their work when given responsibility. The relationship between NSs and supervisor and the daily life in the ward in Norway was different from their home country. Reflection was important for all NSs and they realised that they needed time for this
Nielsen et al. (2020) Norway	To capture the perspectives an experiences of Tanzanian nursing students participating in an international elective	Phenomenological hermeneutical approach	*N* = 16; 4 females, mean age NR, age range 24- 30 years	Sweden Denmark	Different healthcare systems	3–4 weeks	After placement NSs were able to critically reflect about their way of working and approaching patients, became more aware of being competent in taking care of patients with special needs coming from different parts of society and the opportunities and possibilities in a nursing carrier and to make a difference for patients. NSs felt more self-confident. All described that they had been respected and invited to participate in different happenings, socially and professionally. Female NSs felt their life had changed after being abroad
O’Donnell et al. (2022), US	To describe students’ experiences during a 15-week semester involving clinical placement in an Irish university	Qualitative descriptive approach	*N* = 19; gender NR, age NR	Ireland	NR	15 weeks	Understanding formal and informal university environments was expressed as difficult. NSs experienced difficulties being far from home, but became easier as time passed on. The teaching and assessments approaches were different. NSs valued the reflection in guiding personal and professional development and enjoyed meeting the patients and staff, realising that the core values are the same. They also realised that you cannot have culture sensitivity if you do not go abroad to understand what is different
Peel et al. (2021), Australia	To describe experiences, cultural awareness and challenges encountered by final year undergraduate nursing students undertaking a 22-day cultural immersion placement in Nepal	Qualitative descriptive design	*N* = 42; 38 females, gender, age range 21–55 years	Nepal	Hospital	3 weeks	NSs expectations of working alongside the Nepalese NSs was incorrect but when staff got to know them they became more included. The healthcare system was different and the difference between rich and poor, was difficult to relate to. Despite hygienic challenges, NSs acknowledge the innovative nature of Nepalese nurses regarding limited resources. NSs experienced communication difficulties due to language which gave them a unique insight into what it might be like for patients at home. The cultural knowledge gained influenced their experience of culture and cultural expectations
Racine et al. (2021),Canada	To explore the usefulness of international placements in developing culture safety among undergraduate nursing students	Exploratory qualitative design	*N* = 7; 6 females, mean age 24.6 years	Philippines and Uganda	NR	4–6 weeks	Require preparation about the receiving countries’ history, demographics, health problems, and healthcare system. Having some native language capacity was considered critical. The need of being open-minded, self-knowledge about potential racial and cultural biases, being willing to learn and a desire to encounter and accept cultural differences. Being aware of own attitudes and biases and going in with an open attitude to differences and treating people with respect, was considered as having cultural competence
Reid-Searl (2011) Australia	To present the findings of a research which examined the experiences of undergraduate nursing students when undertaking a clinical placement in Thailand	Qualitative exploratory framework	*N* = 8; gender NR, age NR	Thailand	Community, school, orphanages, hospital	4 weeks	NSs were concerned about number of issues such as personal safety, language barriers and missing family and friends, expected to use their skills and to look at nursing from a different cultural perspective. They were concerned that they did not have enough of nursing skills and communication challenges. NSs frequently made comparison between the two health systems. The relationship with peers and the way teachers facilitated the experience was important. Being on exchange NSs became more appreciative of the life at home which influenced attitudes towards their own nursing education and career
Siles Gonzalez (2016) UK	To examine the experience of nursing students on international exchange programmes	Qualitative design	*N* = 7 (from Spain); 4 females, mean age 20.8 years	UK	Hospital	10 weeks	The main problem was the language. NSs felt anxiety and insecurity not understanding the means and ways of working. NSs reported the minor technical training of the lowest bands of nurses in UK and that this was different from where they came from. The high nursing specialization, bands and different levels of nursing surprised them. The nursing role was different. The exchange programme was hard and productive which made them think of the strength and weakness of each system. NSs pointed out the personal and professional growth
Sundal & Ulvund (2023),Norway	To investigate nurses’ experiences after participating in an international clinical placement programme as nursing students while staying for one to three weeks in a paediatric ward	Qualitative study	*N* = 8; gender NR, age NR	Ethiopia	Hospital	3 weeks	NSs learned about unknown medical conditions. They mainly assumed an observer role and performed few nursing procedures. NSs experienced the nurses to always be in a hurry and that the nurses were in a higher hierarchy to them. NSs did not feel they had a role in the ward. Language constituted an obstacle performing nursing procedure and communicating. Limited access to resources led to limited treatment options. NSs expressed that the nurses had a different role
Tjoflåt et al. (2017) Norway	To describe how Malagasy and Norwegian students experienced participating in the two-way exchange programme	Descriptive qualitative study	*N* = 18 (11 Norwegian students, 7 Malagasy students); gender NR, age NR	Madagascar	Hospital	3 months	Norwegian NSs admired the emphasis Malagasy NSs had on technical skills. Malagasy NSs expressed respect about the Norwegian NSs nurse-patient relationship and communication skills. Malagasy NSs expressed clear expectations such as sharing experiences and to learn as much as possible. Several Norwegian NSs did not have any expectations. Both groups were positive towards working together, that they could learn from each other and had different knowledge related to theory and practical skills. There were challenges of working together, such as language barrier, resource scarcity, and how they related to the patients and ethics
Torsvik (2008) Norway	To explore how students developed reflective nursing practice through cultural encounters between students from Tanzania and Norway.	Exploratory study	*N* = 14 (10 Tanzanian students, four Norwegian students); gender NR, age NR	Tanzania	Hospital	3 weeks	There was a positive atmosphere among the two student groups. The Norwegian students experienced the African students as open-minded, warm, friendly, strongly present, singing, laughing and inclusive. The Tanzanian students described the Norwegian students as very co-operative, attentive, with a good approach and interested in adapting to everything. Both groups experienced both similarities and diversity in their roles as nurses. Most differences was found related to nursing care practice where the Norwegian students were impressed about the students from Tanzania and their skills relating to nursing procedures. Tanzanian students feel that the Norwegian students were too concerned with individual patients. There was also a difference in the two groups perceptions of responsibility as a nurse. While the Norwegian students experienced the Tanzanian students the ne less dedicated to the care of patients, the Tanzanian students found the Norwegian students too dedicated to each patient. While the Tanzanian students felt that the Norwegian students communicated too directly to the relatives, the Norwegian students did not experience indirect communication and little empathy as good nursing care.
Ulvund & Mordal (2017) Norway	To investigate how short -term international clinical placement impacted Norwegian nursing students’ development of cultural competency	Qualitative descriptive design	*N* = 18, gender NR, age NR	Ethiopia	Hospital,Health promotion programme	4 weeks	Supervisors played a vital role in making NSs feel safe by providing emotional support and helping them develop an open mind through daily guided reflections. Increased self-esteem supported the NSs’ willingness to care for people from different cultures and to work in different countries. NSs faced language barriers. They learned from being the person who was not being understood and being the person who did not understand. Meetings with people challenged NSs’ empathy, solidarity, and respect. They began to develop interpersonal skills regarding working with people from different cultures
Ulvund et al. (2023), Norway	To investigate nurses' experiences after participating in international clinical placement as students, and how the stay influences their future career as professional nurses	Qualitative design	*N* = 8 (between 2–8 years since the exchange); 8 females, mean age NR, age range 21–30 years	Ethiopia	Hospital	4 weeks	NSs experienced great contrast between Norway and Ethiopia, which increased their cultural awareness, made them aware of immigrant’s challenges and better understanding and knowledge in caring for patients from other cultures. NSs became more open minded after the international experience and more satisfied with the Norwegian health system. Preparation for demanding situations was helpful. Sharing experiences and discussing academic issues was important to cope with the emotional strain and achieve the learning outcomes. Debrief during and after the exchange was valuable

## CRediT authorship contribution statement

**Lasse Andreassen:** Writing – review & editing, Writing – original draft, Methodology, Formal analysis, Data curation, Conceptualization. **Simen A. Steindal:** Writing – review & editing, Supervision, Methodology, Investigation, Data curation. **Jyoti Sarin:** Writing – review & editing, Methodology, Data curation. **Benedicte Sørensen Strøm:** Writing – review & editing, Writing – original draft, Methodology, Formal analysis, Data curation, Conceptualization.

## Declaration of competing interest

The authors declare that they have no known competing financial interests or personal relationships that could have appeared to influence the work reported in this paper.

## Appendix Supplementary materials

Image, application 1
