# Supplementary material for: Undergraduate nursing students’ experiences during international clinical placement: A scoping review
Source: Int J Nurs Stud Adv. 2025 Jul 16;9:100378. doi: 10.1016/j.ijnsa.2025.100378 (PMC12336048; doi:10.1016/j.ijnsa.2025.100378)
Supplement: Supplementary file 1 [file mmc1.docx]

**Appendix 1** Search terms in PubMed

|  | **Search terms** |
| --- | --- |
|  | ‘International clinical experience* OR ‘Cultural competence* OR Cultural competency OR ‘International clinical placement’ OR ‘Study abroad’ OR ‘Education abroad’ OR ‘International education’ OR ‘Student exchange program’ OR ‘overseas stud* OR ‘overseas placement’ OR ‘Exchange Program* OR ‘International Educational Exchange’ OR ‘Global health’ OR ‘World Health’ OR ‘Mobility program* |
| AND |  |
|  | ‘nurse student* OR ‘nursing students’ OR ‘Education, nursing’ OR ‘Students, nursing |
| AND |  |
|  | ‘Student Experiences’ OR ‘Experience* OR ‘Student attitudes’ OR ‘Attitude |
